# Effect of age-based and environment-based cues on reproductive investment in *Gambusia affinis*

**DOI:** 10.1002/ece3.1055

**Published:** 2014-04-01

**Authors:** Eric J Billman, Mark C Belk

**Affiliations:** Department of Biology, Brigham Young UniversityProvo, Utah, 84602

**Keywords:** Cost of reproduction, mosquitofish, phenotypic trajectory analysis, reproductive restraint, reproductive value, terminal investment

## Abstract

We examined the multivariate life-history trajectories of age 0 and age 1 female *Gambusia affinis* to determine relative effects of age-based and environment-based cues on reproductive investment. Age 0 females decreased reproductive investment prior to the onset of fall and winter months, while age 1 females increased reproductive investment as the summer progressed. The reproductive restraint and terminal investment patterns exhibited by age 0 and age 1 females, respectively, were consistent with the predictions from the cost of reproduction hypothesis. Age 0 females responded to environment-based cues, decreasing reproductive investment to increase the probability of overwinter survival and subsequent reproductive opportunities in the following summer. Age 1 females responded to age-based cues, or the proximity of death, increasing investment to current reproduction as future reproductive opportunities decreased late in life. Thus, individuals use multiple cues to determine the level of reproductive investment, and the response to each cue is dependent on the age of an individual.

## Introduction

Patterns of reproductive allocation are influenced by an individual's age and environmental conditions (Fisher 1930; Williams 1966; Roff 2002). Age-based life-history theory predicts patterns of reproductive allocation based on the interaction of age and the probability of survival. The prominent age-based hypothesis, the cost of reproduction hypothesis, predicts the level of reproductive allocation based on future reproductive opportunities assuming that reproduction comes at a cost (e.g., reduction in body condition or survival) that reduces future reproductive opportunities (Williams 1966; Clutton-Brock 1984; Reznick 1985). The cost of reproduction hypothesis predicts that young individuals that have a high reproductive value, or high future reproductive potential (Fisher 1930), should allocate less to current reproduction to ensure future reproductive opportunities. Conversely, old individuals that have low reproductive values should allocate more to current reproduction (i.e., terminal investment; Clutton-Brock 1984), accepting the greater costs of reproduction because future opportunities may not be available (Williams 1966). Reproductive patterns that support the predictions of the cost of reproduction hypothesis have been reported in organisms that have seasonal reproduction (Pärt et al. 1992; Berteaux and Boutin 2000; Descamps et al. 2007) and in short-lived organisms that reproduce multiple times in a single reproductive season (Poizat et al. 1999, 2002; Baker et al. 2008; Creighton et al. 2009).

Environmental conditions can similarly affect the balance of the cost of reproduction trade-off by altering the availability of resources, the rates of physiological processes, or the probability of survival independent of age (McNamara and Houston 1996). Temporal variation in environmental conditions can lead to adaptive life-history responses in organisms to maximize reproduction when environmental conditions are favorable and to conserve energy by reducing or abstaining from reproduction during poor environmental conditions (Winemiller 1993; Ohbayashi-Hodoki and Shimada 2005; Sockman et al. 2006; Lake et al. 2008; Bårdsen et al. 2010). Seasonality can likewise affect patterns of reproductive allocation, particularly if survival through the poor or selective season is dependent on somatic energy storage. Therefore, the pattern of reproductive investment should be affected by environment-based cues that indicate changes in season.

When should an individual be more likely to respond to age-based rather than environment-based cues? We predict that young individuals with high future reproductive opportunities should be more likely to respond to environment-based cues, with greater allocation during favorable environmental conditions, and lower allocation, or reproductive restraint, during poor environmental conditions. Conversely, we predict that old individuals should demonstrate a greater response to age-based cues and terminally invest, that is, high allocation to current reproduction regardless of environmental conditions.

**Figure 1:**
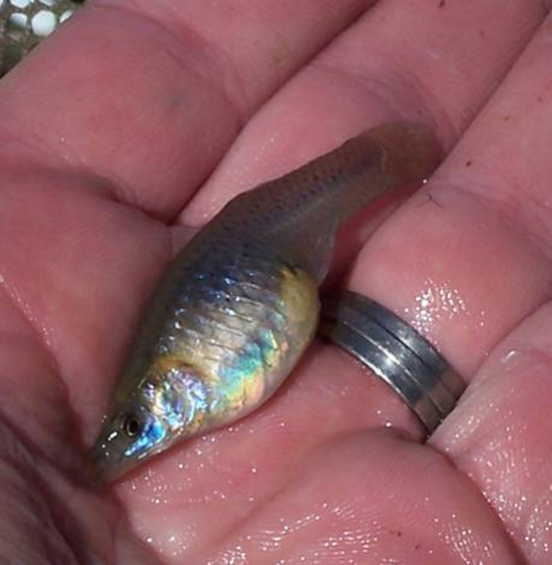
Female *Gambusia affinis*.

We examined reproductive allocation in female *Gambusia affinis* (Fig. 1) from an introduced population in Utah Lake, Utah, USA, to test for patterns consistent with predictions of the reproductive response to age-based and environment-based cues. *Gambusia affinis* provides a good model species to examine life-history responses to both age-based and environment-based cues. *Gambusia affinis* is a live-bearing fish in the family Poeciliidae which is characterized by small-bodied, short-lived fishes. Endemic to southeastern United States, *G. affinis* has been introduced worldwide for mosquito abatement programs (Bay 1972; Rupp 1996; Pyke 2005). In temperate climates, environmental seasonality results in two distinct age classes (age 0 and age 1), both of which are reproductive (Krumholz 1948; Haynes and Cashner 1995; Belk and Tuckfield 2010); all females die before reaching age 2. Because winter acts as a strong selective agent, *G. affinis* must balance age-based and environment-based cues to determine the pattern of reproductive investment (Daniels and Felley 1992). Ideally, we could have followed individuals throughout their lifetime in the natural environment. However, such tracking is not possible in the large lake environment. Rather we used repeated samples of the large population to represent the overall pattern and response to environmental changes in the lake. We view this approach as complementary to controlled experiments that directly test responses to experimental conditions, but do not provide the context of the natural environment. We predict that age 0 females should respond to environment-based cues and exhibit reproductive restraint as the summer progresses, but age 1 females should respond to age-based cues and exhibit a reproductive allocation pattern consistent with terminal investment.

## Methods

### Study site

**Figure 2:**
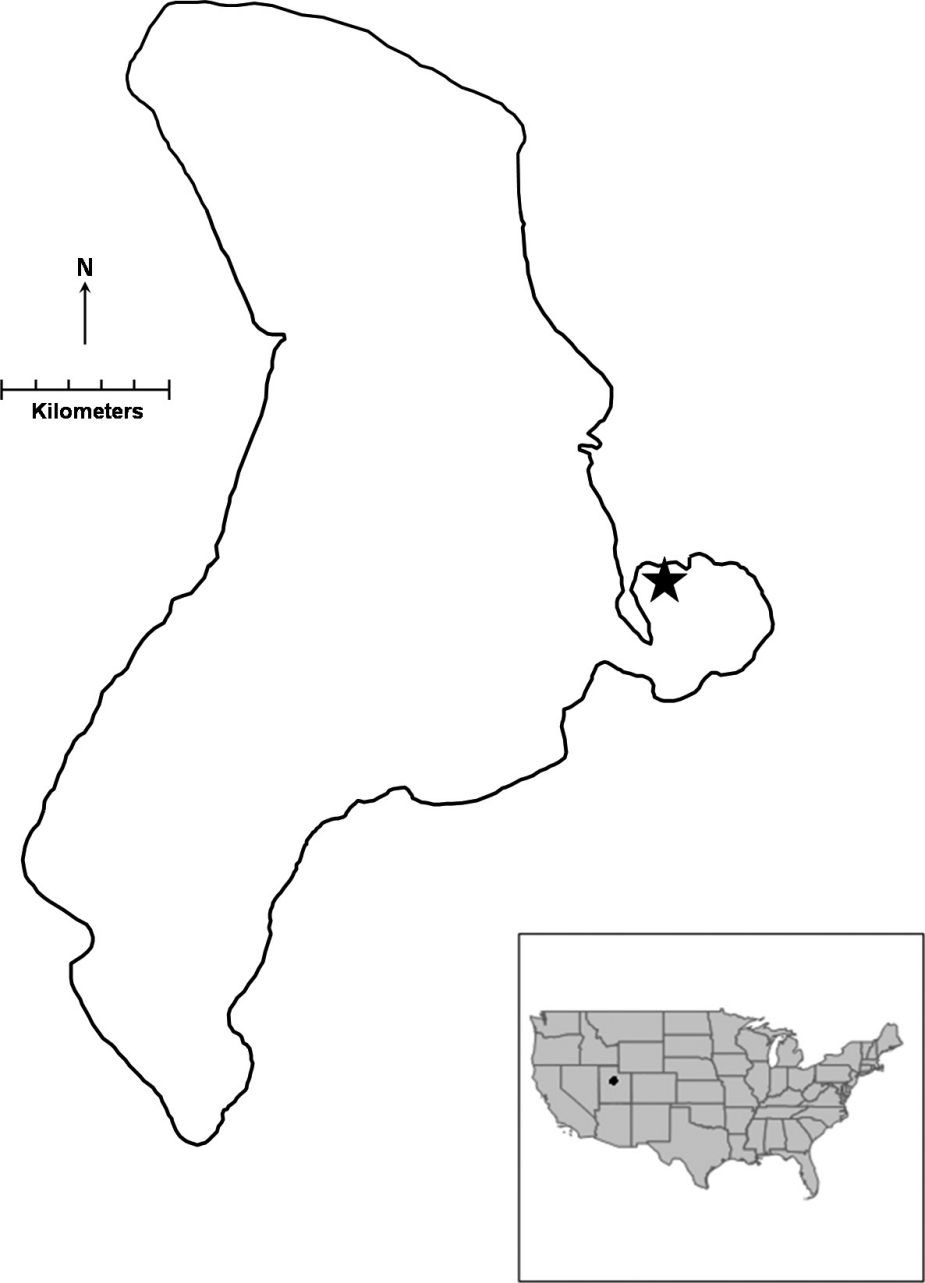
Map of Utah Lake with the star indicating the location of *Gambusia affinis* collections in Provo Bay. Inset shows the location of Utah Lake in Utah, USA.

Utah Lake is a large freshwater remnant of ancient Lake Bonneville in the Great Salt Lake watershed in central Utah (Fig. 2). Utah Lake has a large surface area (∼38,800 ha) but reaches a maximum depth of only about 4 m. Provo Bay is a large (∼1,800 ha) southeast extension of Utah Lake (Fig. 2). It is fringed almost entirely by emergent vegetation including *Scirpus validus*, *Typha latifolia*, and *Phragmites* sp. (Miller and Crowl 2006). *Gambusia affinis* was introduced into Utah in the early 1930s and likely dispersed into Utah Lake between 1934 and 1945 after it was introduced into freshwater springs near the northwest shore of the lake (Rees 1934, 1945). *Gambusia affinis* has since become abundant in the lake. High densities of *G. affinis* occur around the emergent vegetation in Provo Bay where the fish seeks refuge from avian predators and introduced piscivorous fishes (e.g., *Morone chrysops, Micropterus salmonoides*, *Lepomis macrocephalus*, and *L. cyanellus*).

In this temperate environment, *G. affinis* reproduction is strongly seasonal (Krumholz 1948; Hughes 1985; Haynes and Cashner 1995) producing two distinct age classes easily distinguished by body size (Belk and Tuckfield 2010). Summer water temperatures along the margins of Provo Bay range between 15°C and 28°C from the end of April to September (E. J. Billman, unpubl. data.), during which months *G. affinis* are reproductively active. No reproduction and little growth occur during the other 6.5 months of the year because water temperatures are too low (Vondracek et al. 1988; Priddis et al. 2009). Female *G. affinis* survive for only two reproductive seasons in this environment. This results in a distinct size separation between old and young females. Old females (age 1) at the end of their lives reproduce primarily in the early to mid-portion of the reproductive season. Young females (age 0) reproduce from mid-summer to end of the reproductive season determined by decreases in water temperature due to the transition from warm summer to cold winter months. Therefore, age 0 and age 1 females should use different cues to determine the extent of reproductive investment. Age 0 females should base the level of reproductive investment on environmental cues indicating the onset of fall and winter months, while age 1 females should use age-based cues associated with the end of life to determine the level of reproductive investment.

### Population sampling

*Gambusia affinis* were collected under permits from the Utah Division of Wildlife Resources, and collecting procedures were approved by the Institutional Animal Care and Use Committee at Brigham Young University. *Gambusia affinis* were collected along a 1-km section of the north shore of Provo Bay during 2008 and 2009. Samples were collected monthly between the end of April or beginning of May and the middle of September for a total of six samples per summer (Table 1): three samples that consist mainly of age 1 reproductive females (end of April to beginning of July) and three samples that consist mainly of age 0 reproductive females (mid-July to mid-September). Females in each age were classified into three time periods representing early, mid-, and late reproduction (time periods 1, 2, and 3, respectively; Table 1). The first three collections each year represented time periods 1–3 for age 1 females, and the last three collections each year represented time periods 1–3 for age 0 females.

**Table 1:** Summary of collection information for *Gambusia affinis* females in Utah Lake, Utah. Sample sizes indicate the number of females with developing embryos (stage > 2) out of the total number of females (immature and mature) collected

Year	Sample date	Time period: age 0	Time period: age 1	*n* age 0 females	*n* age 1 females
2008	9 May	–	1	0	91/151
	6 June	–	2	0	146/146
	3 July	–	3	0/63	38/39
	18 July	1	–	117/177	6/6
	18 August	2	–	84/218	4/4
	9 September	3	–	31/223	0
2009	28 April	–	1	0	34/75
	29 May	–	2	0	58/58
	26 June	–	3	0/35	43/44
	30 July	1	–	63/92	2/2
	26 August	2	–	74/100	1/1
	17 September	3	–	8/192	1/2

*Gambusia affinis* were collected using large D-nets with 3-mm mesh. We were able to successfully capture fish of all sizes from birth to adults when present. However, fish <18 mm SL were likely underrepresented in our samples because newly born and early developing fish were able to fit through the mesh. These fish represented immature juveniles and would not affect our estimates of reproductively mature adults. Habitat utilized by *G. affinis* (emergent vegetation) precluded using seine nets because there were few areas of open water large enough to use seine nets, and *G. affinis* remained close to emergent vegetation where individuals quickly retreated for refuge. Water depth ranged from 3 to 100 cm where fish were collected. Fish were euthanized with an overdose of tricaine methanesulfonate (MS-222) and immediately placed into 70% ethanol.

Female *G. affinis* from each sample were measured to the nearest mm standard length (SL) and weighed to the nearest 0.1 mg. Sex of fish less than 13 mm SL could not be determined. Females were dissected to determine pregnancy. Ovaries were removed and weighed to the nearest 0.1 mg. Additionally, the eviscerated bodies of the females were weighed to the nearest 0.1 mg. The number of embryos in the ovaries for each individual was counted, and the stage of development of the embryos was determined according to Haynes (1995). Females and their clutches were dried for 24 h at 55°C, after which they were weighed to the nearest 0.1 mg. Female size at maturity was determined as the size (SL mm) at which >50% of females contained developing embryos (Johnson and Belk 2001), defined as stage 3 or greater according to Haynes (1995).

### Statistical analyses

We used three life-history traits to characterize life history for reproductive females in both age classes: (1) reproductive allotment, (2) clutch size, and (3) offspring dry mass. Reproductive allotment is the clutch dry mass and represents the reproductive investment of a female to her current clutch of offspring (Johnson and Belk 2001; Scott and Johnson 2010). Clutch size equals the total number of developing embryos in the female. Offspring dry mass equals the per capita dry weight of the developing offspring (clutch dry mass divided by clutch size).

### Univariate analyses

We used a mixed model analysis of covariance (ANCOVA; Proc Mixed; SAS Institute, Inc. 2008) to test for differences between age 0 and age 1 females for each of the three life-history traits. Restricted maximum likelihood was used to fit the model. The random variable in the model for each analysis was the year of collection (i.e., 2008 and 2009). The main effects in the model were age, time period, and their interaction. For each analysis, female dry mass (log_10_-transformed) was the covariate in the model; additionally, embryonic stage of development (hereafter stage) was used as an additional covariate in the analyses for reproductive allotment and offspring dry mass. Prior to analyses, we transformed life-history traits (log_10_-transformed) to accommodate for potential nonlinear relationships between variables. A Tukey's test was used for post hoc mean comparisons for all three tests.

### Phenotypic trajectory analysis

Collyer and Adams (2007) and Adams and Collyer (2009) recently described a general framework to analyze multivariate phenotypic trajectories in evolutionary studies that has successfully been used in life-history studies (Chun et al. 2007; Dennis et al. 2011). In studies of life-history evolution, the multivariate life-history strategies of evolutionary groups across multiple time periods can be described by a trajectory in multivariate life-history trait space. Attributes of life-history trajectories (i.e., magnitude of phenotypic change, direction of phenotypic change, and shape of the trajectory) can be statistically compared to determine the extent to which life-history strategies are parallel, convergent, or divergent. Additional, life-history trajectories can be compared to age-based life-history predictions to determine the extent to which lifetime life-history strategies support life-history theory.

Life-history traits must be transformed prior to life-history trajectory analysis to account for variation in traits due to embryonic stage of development and female dry mass. Clutch characteristics for lecithotrophic species (i.e., clutch dry mass and offspring dry mass) will be negatively correlated with stage of development (Marsh-Matthews et al. 2005). *Gambusia affinis* has been described as a strict lecithotrophic species, although it has recently been reported that *G. affinis* exhibits maternal provisioning typical of matrotrophic species (Marsh-Matthews et al. 2005). However, this maternal provisioning is not sufficient to preclude loss of mass across the entire gestation of the embryos as demonstrated in this study (Table 2). Because mass loss accumulates with embryonic stage of development, we used the coefficients for stage from the ANCOVA models for reproductive allotment and offspring dry mass to adjust these life-history traits (log_10_-transformed) to stage 3 for each individual. To account for the positive relationship between life-history traits and female dry mass, we regressed each life-history trait (log_10_-transformed) separately on female dry mass (log_10_-transformed) with the slope constrained to one. By constraining the slope, we account for isometric changes in life-history traits but maintain allometric patterns of reproductive restraint (hypoallometric changes in life-history traits) and terminal investment (hyperallometric changes in life-history traits) if they are present. The residuals of these regressions are used as life-history variables for the life-history trajectory analysis.

**Table 2:** Analysis of covariance tables for mixed models comparing reproductive allotment, clutch size, and offspring dry mass for female *Gambusia affinis* as a function of age, time period, and the covariates female dry mass and stage of embryonic development

Effect	Num DF	Den DF	*F*-statistic	*P*-value
Reproductive allotment				
Age	1	773	10.86	0.010
Time period	2	773	22.22	<0.001
Female dry mass	1	773	424.56	<0.001
Stage	1	774	25.97	<0.001
Age × time period	2	773	88.11	<0.001
Clutch size				
Age	1	775	49.97	<0.001
Time period	2	774	46.69	<0.001
Female dry mass	1	775	152.76	<0.001
Age × time period	2	775	68.92	<0.001
Offspring dry mass				
Age	1	773	4.28	0.039
Time period	2	773	84.76	<0.001
Female dry mass	1	773	113.01	<0.001
Stage	1	774	9.72	0.002
Age × time period	2	773	5.96	0.003

Additionally, we generated four reference trajectories based on predictions of allocation due to age-based and environment-based cues and the life-history trade-off between offspring size and number. Two trajectories represented life-history strategies that reflect a change in reproductive investment due to age-based cues (i.e., increased allocation to current reproduction with increase in time period; terminal investment). The other two trajectories represented life-history strategies that represent predictions of reproductive investment due to environment-based cues (i.e., increased allocation to future reproduction with increase in time period; reproductive restraint). For each pair of trajectories, one trajectory represented a life-history strategy with greater allocation to offspring size (i.e., offspring dry mass), while the other represented a strategy with greater allocation to offspring number (i.e., clutch size).

We calculated principal component scores for each individual (including reference data) from a principal components analysis performed on a correlation matrix with life-history traits (i.e., residuals generated above) as the dependent variables. Principal component scores were used as response variables in a mixed model multivariate analysis of variance (MANOVA; Proc Mixed; SAS Institute, Inc. 2008) to assess differences in life-history strategy of age 0 and age 1 females. Year of collection was the random effect in the model. In addition to the main effects of age and time period, we included an index variable that accounts for the ordered nature of the principle components (i.e., PC1, PC2, PC3). Principle components are orthogonal; therefore, the magnitude and direction of differences between levels of main effects on one principal component have no bearing on the magnitude and direction of differences between levels on the other principal components. We included all interactions of the main effects in the MANOVA. The interactions of the index variable with main effect(s) test differences in the levels of the main effect(s) while allowing the magnitude and direction of differences to vary independently among principal components; these interactions are the terms of interest in the MANOVA (Rencher 2002; Butler et al. 2009; Wesner et al. 2011; Hassell et al. 2012).

Following a significant interaction of age, time period, and index variable, we applied the phenotypic trajectory analysis. Differences in trajectory attributes were calculated and statistically tested using a residual randomization approach (Adams and Collyer 2007). The residuals from a reduced model of the MANOVA that lacked the age, time period, and index variable interaction were randomized and added to the predicted values to produce a random data set. We then used the full MANOVA to analyze the random data set. This procedure was repeated 9,999 times to generate a distribution of random differences of trajectory attributes to compare to the observed differences in trajectory attributes. The phenotypic trajectory analysis was conducted in R (R Core Development Team 2010); mixed model MANOVAs were conducted in ASREML-R version 3.00 (Butler et al. 2009) within R.

## Results

**Figure 3:**
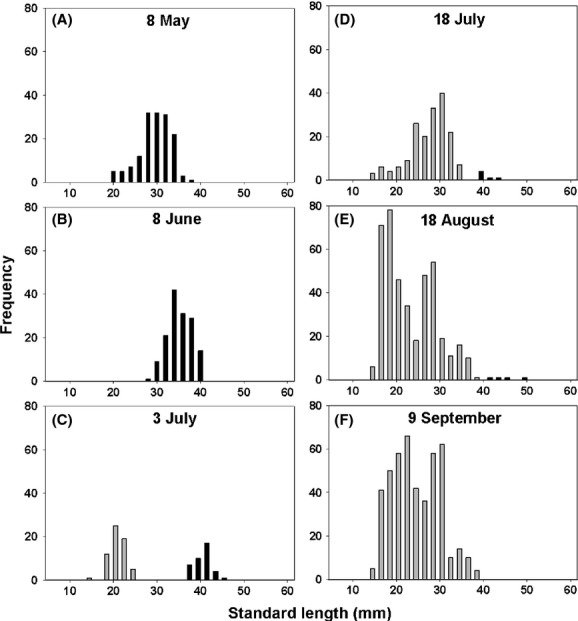
Length histograms of *Gambusia affinis* in Provo Bay of Utah Lake, Utah, for the six collecting periods in 2008. Black bars indicate age 1 females, and gray bars indicate age 0 females.

Density of *G. affinis* was low in early summer representing the few females and males that had successfully overwintered. Density increased after the first cohort of age 0 fish was born in June; densities increased dramatically through the summer as additional clutches were produced by both age 0 and age 1 females. This pattern in population density resulted in few fish sampled in early summer samples, and many more fish sampled in later samples (Table 1). Growth patterns of female *G. affinis* as determined by length-frequency histograms were similar in both summers and resulted in two distinct age classes, that is, >4 mm separating age classes in all samples (2008 collections shown in Fig. 3). Size at maturity for females in this population was determined for time periods 1 and 2 for age 0 females (mid- to late July and August); in time period 3, there was not a size for which greater than 50% of females were pregnant, precluding the ability to determine size at maturity for this time period. For time periods 1 and 2 across both years, the estimated size at maturity was consistently 25 mm SL. In time period 1 for age 1 females, a low percentage of females were pregnant (63% and 41% for time period 1 in 2008 and 2009, respectively). However, all or nearly all (>97%) of age 1 females were pregnant in time periods 2 and 3 in both years. For age 0 females at or above the estimated size at maturity, the percentage of pregnant females was high for time periods 1 and 2 for both years (93–96% and 89–93% in 2008 and 2009, respectively); however, the percentage of pregnant females declined greatly in time period 3 to 32% in 2008 and 15% in 2009.

**Figure 4:**
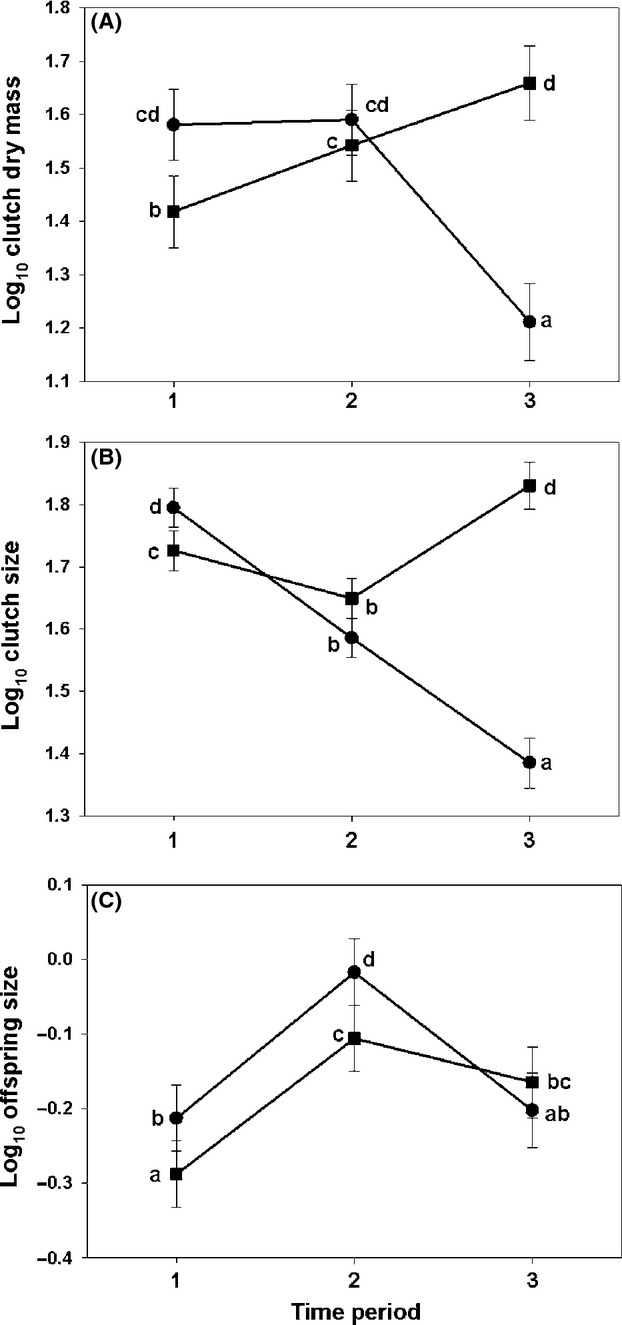
Least squares means (±SE) of life-history traits for female *Gambusia affinis* in Provo Bay of Utah Lake, Utah: (A) reproductive allotment (clutch dry mass; mg), (B) clutch size, and (C) offspring dry mass (mg). Circles represent age 0 females, and squares represent age 1 females. Means have been adjusted to account for inherent differences in female body size between the two ages. Different letters indicate significant (*α* = 0.05) differences in means.

Reproductive allotment was significantly affected by age, time period, and their interaction after controlling for variation due to female dry mass and embryonic stage of development (Table 2). Age 0 females had relatively high reproductive allotment for the first two time periods, but reproductive allotment significantly declined in the third time period (Fig. 4A). For age 1 females, reproductive allotment significantly increased with each time period. However, reproductive allotment at time period 3 for age 1 females was not significantly different than reproductive allotment for age 0 females in the first two time periods (Fig. 4A). Reproductive allotment was positively related to female dry mass (coefficient = 1.153; *P* < 0.001), but negatively related to embryonic stage of development (coefficient = −0.021; *P* < 0.001).

Clutch size was significantly affected by age, time period, and their interaction after controlling for variation due to female dry mass (Table 2). For age 0 females, clutch size significantly decreased with each time period (Fig. 4B). Age 1 females also had a significant but smaller decrease in clutch size from time period 1 to time period 2; however, clutch size significantly increased from time period 2 to time period 3 such that clutch size at time period 3 was the largest for age 1 females (Fig. 4B). Clutch sizes for age 0 females at time period 1 and age 1 females at time period 3 were not significantly different. Clutch size was positively related to female dry mass (coefficient = 0.587; *P* < 0.001).

Offspring dry mass was significantly affected by age, time period, and their interaction after controlling for variation due to female dry mass and embryonic stage of development (Table 2). Both age 0 and age 1 females had a similar pattern of small offspring at time period 1 with a significant increase in offspring dry mass at time period 2 (Fig. 4C). For age 0 females, offspring dry mass was reduced in time period 3 and was not significantly different than offspring dry mass at time period 1. For age 1 females, offspring dry mass was not significantly different between time periods 2 and 3 (Fig. 4C). The largest offspring were produced by age 0 females in time period 2. Offspring dry mass was positively related to female dry mass (coefficient = 0.500; *P* < 0.001) and negatively related to embryonic stage of development (coefficient = −0.011; *P* = 0.002).

**Figure 5:**
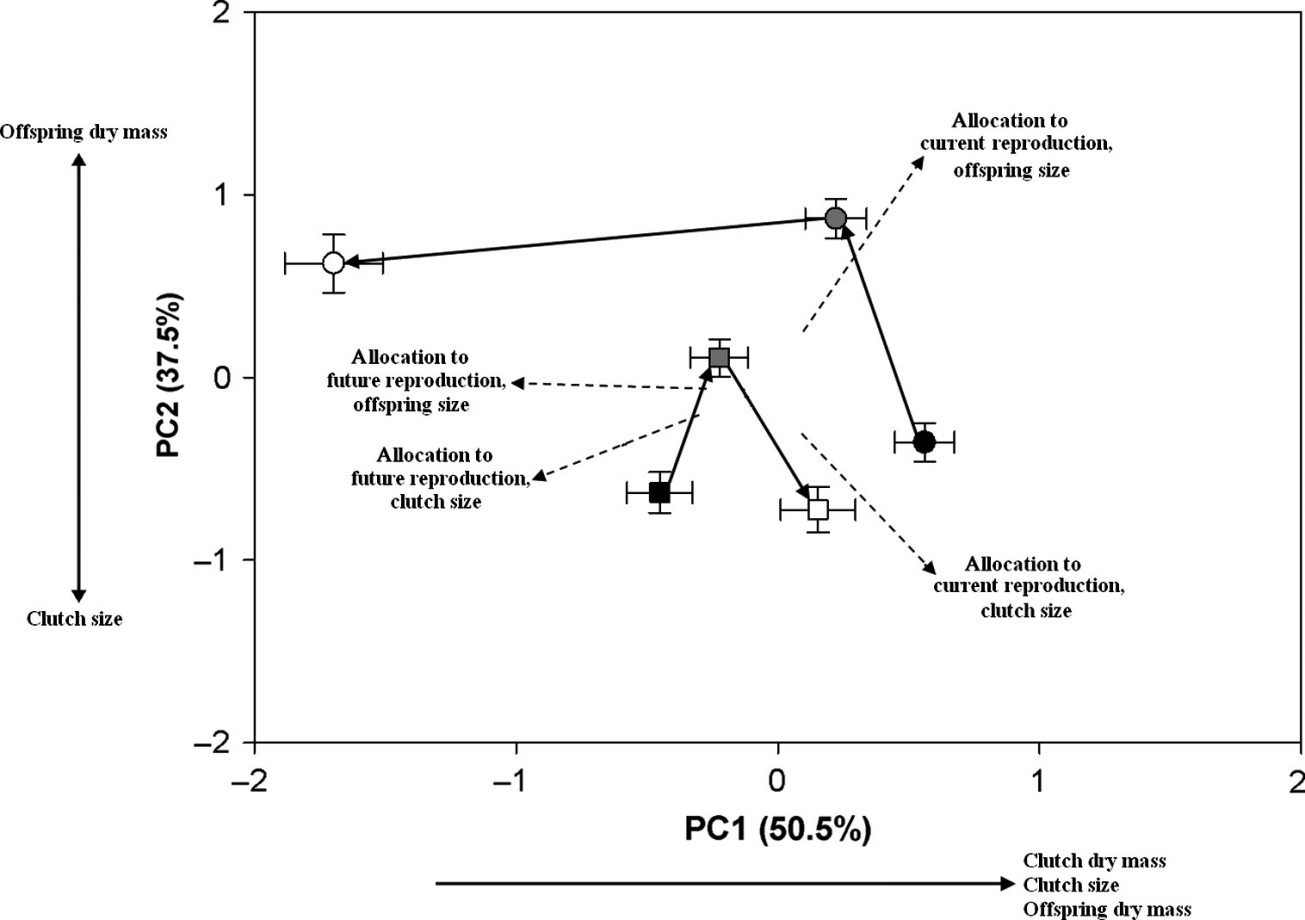
Least squares means (±SE) of principal component scores for life-history trajectories defined by three time periods for age 0 (circles) and age 1 (squares) female *Gambusia affinis*. Black symbols = time period 1; gray symbols = time period 2; open symbols = time period 3. Axes are scaled according to the amount of variation explained by principal components (J. Rohlf, pers. comm.).

We found significant differences in the multivariate life-history trajectory between ages (age by index variable interaction) and among time periods (time period by index variable interaction), as well as in their interaction (age by time period by index variable; Table 3). The significant three-way interaction suggested that there were significant differences in one or more life-history trajectory attributes between the trajectories for age 0 and age 1 females. Life-history trajectories had significant differences in the magnitude of phenotypic change (*MD*_1,2_ = 1.204; *P*_size_ < 0.001) as age 0 females exhibited a greater amount of life-history change compared with age 1 females (Fig. 5). The direction of the trajectories was significantly different between the ages (*θ*_1,2_ = 117.45°; *P*_*θ*_ = 0.003). Age 0 females followed a pattern of change consistent with increased allocation to future reproduction, while age 1 females followed a trajectory of increased allocation to current reproduction (Fig. 5). Finally, the shape of the trajectories was significantly different (*D*_Shape_ = 1.470; *P*_Shape_ < 0.001), mainly in the balance of the trade-off between offspring size and number. Age 0 females initially allocated more to offspring number but switched to greater allocation to offspring size compared with offspring number in time periods 2 and 3 (Fig. 5). Age 1 females switched from greater allocation to offspring number at time period 1 to greater allocation to offspring size at time period 2; in time period 3, age 1 females had greater allocation to offspring number (Fig. 5).

**Table 3:** Multivariate analysis of variance table for the mixed model comparing the life-history strategy (defined by principal components derived from three life-history traits) of female *Gambusia affinis* as a function of age, time period, and an index variable (accounts for ordering of principal components; see Methods for explanation)

Effect	Degrees of Freedom	Wald statistic	*P*
Age	1	772.62	<0.001
Time period	2	199.83	<0.001
Index variable	2	0.09	0.993
Age × time period	2	88.92	<0.001
Age × index variable	2	9.84	0.007
Time period × index variable	4	354.76	<0.001
Age × time period × index variable	4	204.93	<0.001

## Discussion

In this study, we documented how the interaction of age-based and environment-based cues affect the pattern of reproductive investment in *G. affinis*. The comparisons of age 0 and age 1 multivariate life-history trajectories to reference trajectories demonstrated that age 0 females followed a pattern of decreased investment to current reproduction, or reproductive restraint. On the other hand, age 1 females followed a pattern similar to the reference trajectories indicating an increase in the level of investment to current reproduction, or terminal investment. Age 0 females demonstrated a pattern of reproductive restraint in late summer in response to environment-based cues indicating the onset of winter. This life-history response likely increases a female's probability of overwinter survival; therefore, age 0 females demonstrated a shift toward allocation to future reproduction. For age 1 females, the extent of reproductive investment across time periods was consistent with predictions from age-based life-history theory: females exhibited increased allocation to current reproduction (i.e., terminal investment) as summer progressed.

The patterns of reproductive investment in age 0 female *G. affinis* demonstrated a shift in the balance of fitness benefits of reproductive investment to current reproduction and the costs to future reproduction (Williams 1966). This change in life-history strategy is consistent with predictions of the cost of reproduction hypothesis; however, the change in costs and benefits is driven largely by environment-based cues rather than age-based cues. By exhibiting reproductive restraint late in summer, age 0 females should be able to increase their probability of overwinter survival, thus increasing future reproductive potential. The long winter period in central Utah (at least 7 months) acts as a strong selective agent for *G. affinis* such that only individuals with sufficient somatic storage will be able to overwinter. The amount of somatic storage necessary for an individual to overwinter increases with the length of winter (Schultz and Conover 1997), and the rate of storage depletion is inversely related to size (Schultz and Conover 1999). Therefore, females that exhibit reproductive restraint late in the summer and allocate more energy to growth and somatic storage will increase their probability of overwinter survival. Reznick and Braun (1987) documented a similar reduction in reproductive investment in age 0 *G. affinis* in late summer in a population at the northern extent of the species' native range; this reduction in reproduction corresponded to an increase in somatic storage. Similar patterns of reproduction and somatic storage have also been documented in *G. holbrooki*, the sister species of *G. affinis* (Meffe and Snelson 1993; Pérez-Bote and López 2005). A reduction in either clutch size or reproductive investment based on environmental cues has been documented in other fish species (Hatch and Elias 2002; Fox et al. 2011), birds (Sockman et al. 2006), and mammals (Bårdsen et al. 2010).

Despite the reproductive restraint exhibited late in summer, age 0 females had a high level of reproductive investment in the first two time periods similar to the extent of reproductive investment in age 1 females (Table 2; Fig. 4A). Age 0 females appear to be allocating more to current reproduction in the first two time periods and then switching to allocation to future reproduction by time period 3. An alternative strategy for age 0 females that we might predict from age-based life-history theory would be a strategy with low investment or abstinence during all time periods to grow and store energy. This alternative strategy would allow females to have an increased probability of overwinter survival and to benefit from increased reproductive potential as age 1 fish due to their larger body size. However, patterns of reproductive investment observed in age 0 females suggest that winter mortality is high regardless of body size. By increasing reproductive investment early in the summer, an age 0 female increases the probability that at least some of her offspring, if not herself, will survive the winter to reproduce in the following summer. That there is high winter mortality is evident in the greatly reduced densities of *G. affinis* in April compared with the previous September (based on catch per unit effort; E. J. Billman unpubl. data.).

As summer progresses and winter approaches, the reproductive value of newly born offspring should decline due to winter mortality; the multivariate life-history strategy exhibited by age 0 females reflects this reduction. Depletion of somatic storage occurs more rapidly in small fish leading to size-dependent winter mortality (Schultz and Conover 1999). In this population, the smallest individual (female or male) collected in the first collection for both years (9 May 2008 and 28 April 2009) was 18 mm SL, indicating a potential minimum size for overwinter survival. For the first two time periods, age 0 females shifted the balance of the offspring size versus number trade-off; age 0 females had large clutches of poorly provisioned embryos in time period 1 and smaller clutches of well-provisioned embryos in time period 2 (Fig. 4B and 3C). This shift in the balance of the trade-off is likely an adaptive strategy: females have many small offspring early when individuals have sufficient time to reach a minimum size for winter survival and switch to having fewer larger offspring later. We predict that the switch to better provisioned offspring at time period 2 increases the probability that juveniles will reach the minimum size for winter survival despite the shorter time until the onset of this selective season (Heins et al. 2004). Similarly, we predict that offspring born in late summer will have insufficient time to reach the minimum size with enough somatic storage to survive winter, will have a low reproductive value (Sockman et al. 2006), and will not likely contribute to the mother's lifetime fitness (i.e., representation in subsequent generations). The pattern of reproductive restraint in time period 3 by age 0 females reflects the marginal returns of offspring born late in the season.

Reproductive investment by age 1 *G. affinis* females was consistent with predictions of the cost of reproduction hypothesis. As the summer progressed, these females increased reproductive investment characteristic of individuals that are terminally investing (Clutton-Brock 1984). At the beginning of the reproductive season, *G. affinis* females utilize remaining somatic storage for the first reproductive bout (Reznick and Braun 1987). The low reproductive investment of age 1 females and high proportion of nonreproductive females suggests that few somatic stores remain after the long winter season. Alternatively, these females might be demonstrating reproductive restraint to allocate more energy to growth to receive the increase in reproductive output afforded by a larger body size. Belk and Tuckfield (2010) also reported evidence demonstrating that reproductive allocation in *G. affinis* is consistent with predictions from the cost of reproduction hypothesis. In their study, age 1 females had higher reproductive allocation compared with age 0 females, and consequently experienced a significant decline in escape performance, evidence that older females experienced a greater cost of reproduction due to higher reproductive allocation (i.e., terminal investment).

The results of this study demonstrated that *G. affinis* females determine the level of their reproductive investment based on age and environmental cues. However, alternative mechanisms might generate or contribute to the observed patterns. We assumed that the two ages observed in this population were sequential cohorts. Alternatively, the population could consist of two distinct life-history phenotypes that represent a short, fast reproductive life and a long, slow reproductive life (Roff 2002; Belk and Tuckfield 2010). Age 0 females had high reproductive allocation in time periods 1 and 2 that was similar to reproductive allocation by terminally investing age 1 females, a pattern that seems consistent with the difference in mortality and expected lifespan if two life-history phenotypes existed (Reznick and Endler 1982; Johnson and Belk 2001). However, we would expect that the age 0 cohort would include both reproductive and nonreproductive individuals in each time period if there were two life-history phenotypes. In this study, the vast majority (>89%) of age 0 females larger than 24 mm SL had developing embryos in time periods 1 and 2; those without developing embryos were primarily small females that had just reached reproductive maturity (25–27 mm SL). Fewer age 0 females had developing embryos in time period 3, a pattern that is expected as females reduce and cease reproduction due to environment-based cues indicating the end of summer. Therefore, the data in this study are not consistent with predictions for a population with two life-history phenotypes.

Seasonal variation in resource availability provides another explanation for the differences in reproductive allocation observed between age 0 and age 1 females. Resource availability necessarily affects rates of resource acquisition which in turn affects the amount of energy available for allocation into competing demands. Therefore, rates of resource acquisition provide more or less energy for investment into both current and future reproduction resulting in changes in investment that can occur without changing the proportional allocation (Winemiller 1993; Reznick et al. 2000; Jennions et al. 2006; Belk and Tuckfield 2010). For age 1 females, resource availability may increase from April to June, while for age 0 females, resource availability may decrease with the onset of fall and winter months. Under this resource availability scenario, we would predict reproductive investment to increase for age 1 females and decrease for age 0 females as was observed in this study. However, Vondracek et al. (1988) demonstrated that larger (older) females allocated increasingly more of their available energy to reproduction and less to growth compared with smaller (younger) females regardless of the level of resource availability. Additionally, Reznick and Braun (1987) demonstrated that age 0 females allocated more energy to somatic storage and less to reproduction in fall despite a decline in resource availability. Thus, the life-history strategies observed in age 0 and age 1 females are not consistent with the pattern expected from seasonal resource availability alone.

In this study, we were unable to follow individual females, but instead used population means for each age to characterize life-history trajectories. While this methodology is the standard for life-history evolution studies of poeciliids (Reznick and Braun 1987; Daniels and Felley 1992; Johnson and Belk 2001; Jennions et al. 2006), it necessarily assumes that individuals of each age have the same condition or state, that is, they have had the same reproductive history and have the same future reproductive potential (McNamara and Houston 1996). Because poeciliids can have multiple clutches in a reproductive season, age classes that are established on an annual basis will include individuals that have large variation in birth date. This pattern can be observed in the size distribution of overwintered females in the April and May samples (Fig. 3). Age 1 females in April and May include mature females that had reproduced the previous year as well as females that have not yet reached reproductive maturity. Given this variation in age 1 females, we would predict that small overwintering females would live longer and reproduce more times than large overwintering females (Haynes and Cashner 1995). This does not invalidate the results of our study; we still predict that small overwintering females will determine the extent of reproductive investment given age-based cues considering there is no evidence that female *G. affinis* in this population live to age 2. The effect of variation in birth date within age 0 and age 1 females on the observed multivariate life-history trajectories could be further examined with an extensive mark–recapture study. Females could be captured and marked according to size multiple times across two reproductive seasons to determine growth and survival patterns. This would not only verify patterns of overwinter survival but could also determine how birth date affects patterns of reproductive investment.

In this study, we observed patterns in multivariate life-history trajectories of two age classes of *G. affinis* demonstrating that females use multiple cues to determine the level of reproductive investment. For this population of *G. affinis*, patterns in multivariate life-history trajectories indicated that the shift in reproductive investment (positive or negative) as a response to age-based and environment-based cues is dependent on the age of the female. This demonstrates the importance of examining multiple cues in life-history studies to determine how cues interact across an organism's lifetime (Fisher 1930; Cotter et al. 2011).
